# Klebsiella pneumoniae Prostatic Abscess Complicated by Septic Pulmonary Emboli in a Patient With Uncontrolled Diabetes Mellitus

**DOI:** 10.7759/cureus.117909

**Published:** 2026-10-14

**Authors:** Adel Kenawi, Owais Ahmad, Mohammad Humeda, Ujala Razaq, Shah Nawaz

**Affiliations:** 1 Cardiology, Tameside and Glossop Integrated Care NHS Foundation Trust, Ashton-under-Lyne, GBR; 2 Internal Medicine, Tameside and Glossop Integrated Care NHS Foundation Trust, Ashton-under-Lyne, GBR; 3 General Medicine, Tameside and Glossop Integrated Care NHS Foundation Trust, Ashton-under-Lyne, GBR; 4 Gastroenterology, Tameside and Glossop Integrated Care NHS Foundation Trust, Ashton-under-Lyne, GBR

**Keywords:** klebsiella pneumoniae (kp), metastatic klebsiella syndrome, prostatic abscess, pulmonary embolism (pe), septic pulmonary embolism

## Abstract

Prostatic abscess is an uncommon complication of bacterial infection that may be associated with bacteremia and metastatic infection, particularly in patients with diabetes mellitus. A 47-year-old man with poorly controlled diabetes presented with fever and systemic illness. His white blood cell count was 26 × 109/L, and C-reactive protein (CRP) concentration was 348 mg/L; blood cultures grew Klebsiella pneumoniae. Persistent illness after five days of intravenous antimicrobial therapy prompted abdominal and pelvic imaging. The CT suggested a prostatic abscess, and a pelvic MRI demonstrated extensive intraprostatic collections with near-complete replacement of the normal prostatic architecture. Right-sided pyelonephritis and a small right perirenal collection were also identified. Subsequent dyspnea and a new oxygen requirement prompted thoracic imaging, which demonstrated multiple bilateral pulmonary nodules, including cavitating lesions. These findings were considered consistent with probable septic pulmonary emboli in the setting of a deep genitourinary infection. Echocardiography was not completed before transfer, limiting exclusion of an endocardial source. Following prostatic drainage, intravenous antibiotic therapy, and management of hyperglycemia, the patient improved clinically. Follow-up pelvic MRI demonstrated resolution of the prostatic abscess. This case highlights the importance of investigating an occult abscess when systemic infection persists despite antimicrobial therapy and considering septic pulmonary embolism when new cavitating pulmonary nodules accompany bacteremia.

## Introduction

Prostatic abscess may be difficult to distinguish clinically from acute bacterial prostatitis. Diabetes mellitus is an important predisposing condition, and persistent systemic illness despite antimicrobial therapy warrants investigation for an abscess [1-3]. *Klebsiella pneumoniae* can cause prostatic abscess and bacteremia; invasive infections may also be associated with distant infectious foci [4,5].

Septic pulmonary embolism (SPE) results from infected embolic material entering the pulmonary circulation. The CT findings may include peripheral nodules, cavitation, pleural-based opacities, and a feeding-vessel sign [6-8]. Clinical context and assessment for potential infectious sources are essential to interpretation [9,10]. A previous report described *K. pneumoniae* prostatic abscess with SPE and meningitis [11]. We describe an extensive prostatic abscess with associated renal infection and pulmonary findings suggestive of SPE, while acknowledging uncertainty about the precise embolic source.

## Case presentation

A 47-year-old man with poorly controlled diabetes mellitus presented with high-grade fever and systemic illness. Initial investigations showed marked inflammatory abnormalities, with a WBC count of 26 × 10⁹/L (reference range: 4-10 × 10⁹/L) and a C-reactive protein (CRP) concentration of 348 mg/L (reference range: ≤5 mg/L). Blood cultures grew *K. pneumoniae*. He received intravenous antimicrobial therapy but remained clinically unwell after five days, prompting investigation for an occult infectious focus.

A CT of the abdomen and pelvis demonstrated an enlarged prostate containing multiple hypodense areas with surrounding pelvic fat stranding (Figure 1). Subsequent pelvic MRI demonstrated extensive intraprostatic abscess formation, with near-complete replacement of the normal prostatic architecture (Figure 2). Abdominal imaging also showed right-sided pyelonephritis and a small right perirenal collection.

**Figure 1:**
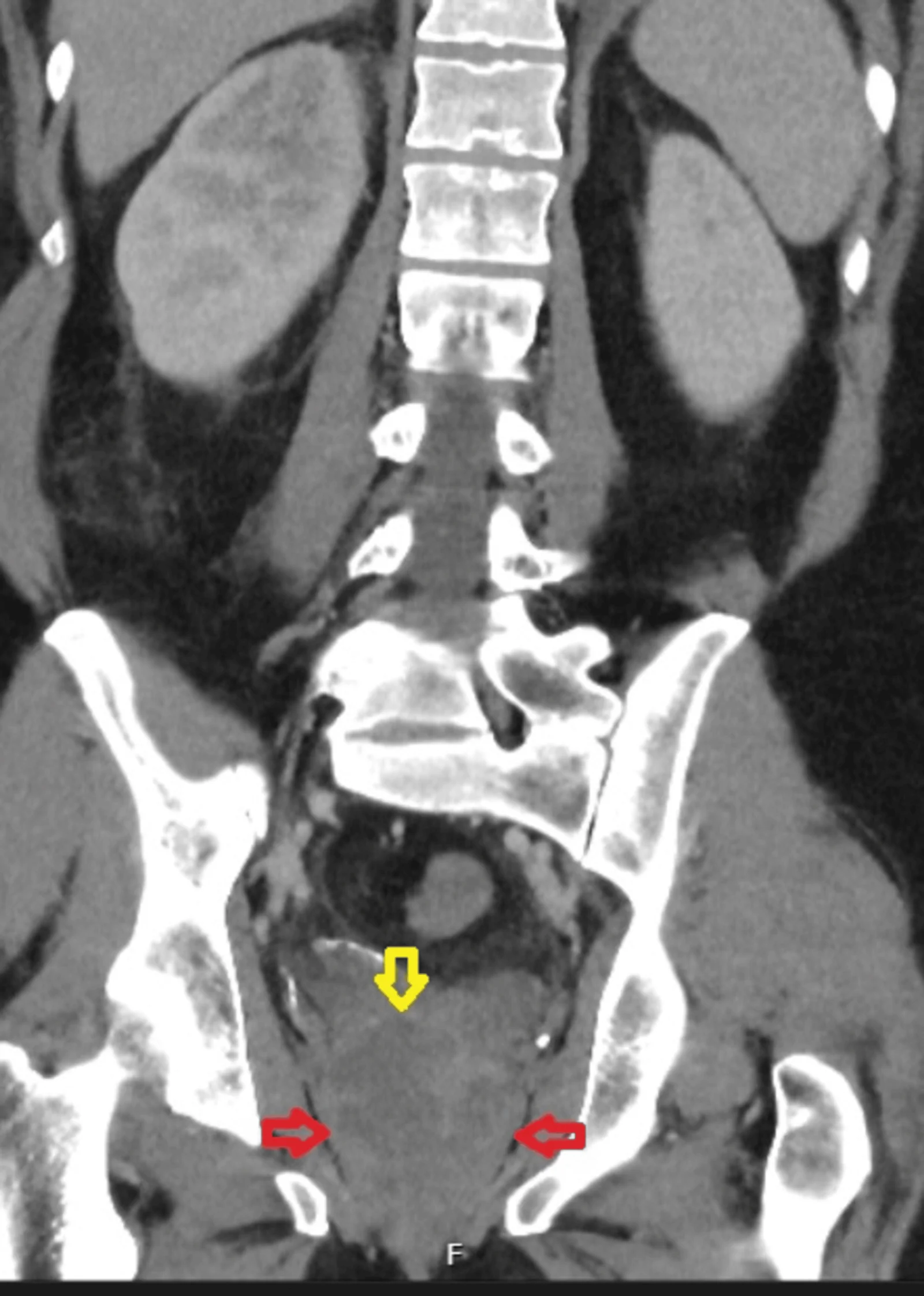
CT abdomen and pelvis showing enlarged prostate (red arrows) and areas of necrosis inside the enlarged prostate (yellow arrow)

**Figure 2:**
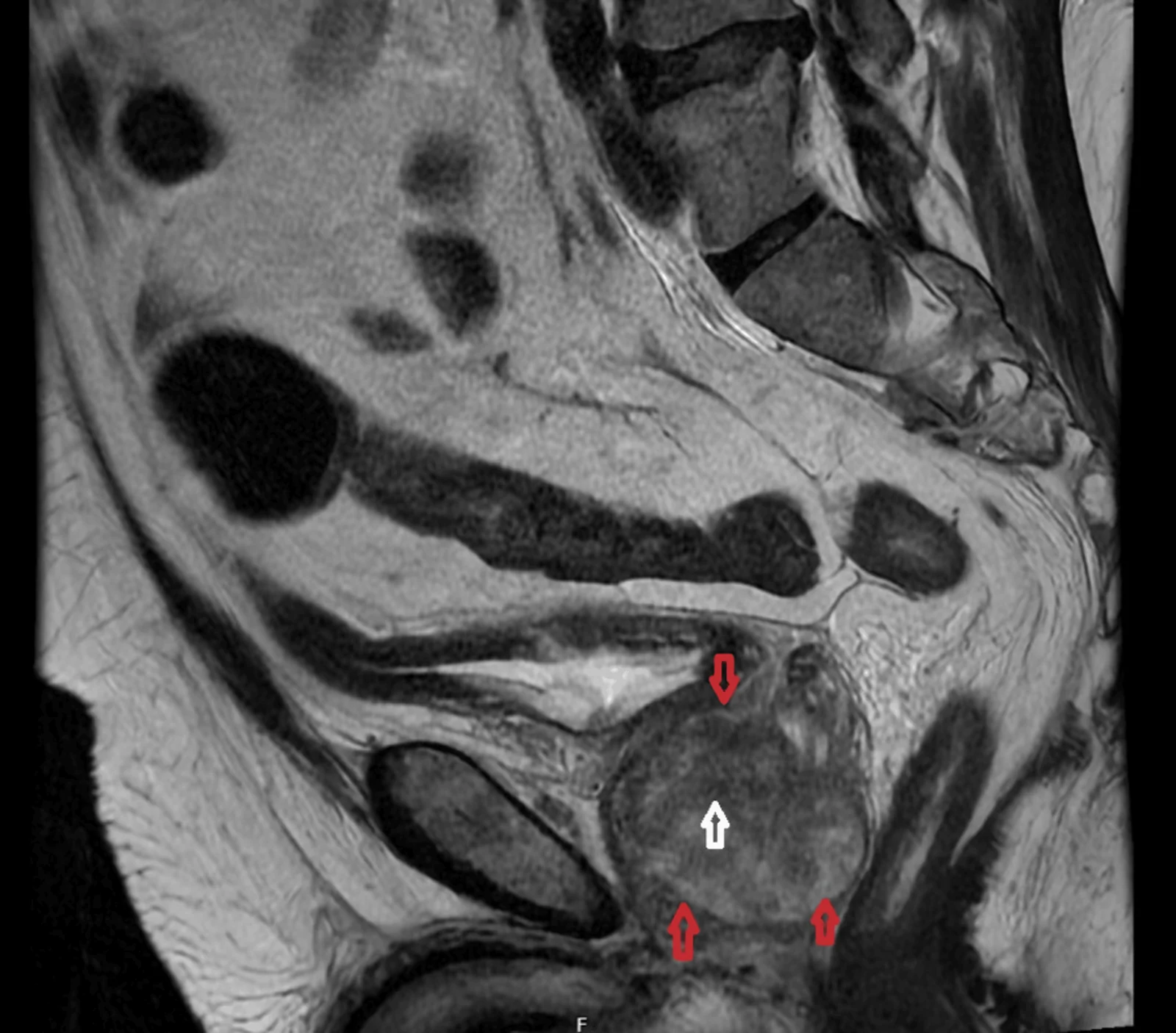
Pelvic MRI showing extensive intraprostatic collections and marked distortion of the normal prostatic architecture (red arrows). The white arrow indicates an internal fluid-containing component.

During hospitalization, the patient developed dyspnea and required supplemental oxygen at 2 L/min. A chest radiograph obtained approximately three days earlier had not shown obvious large pulmonary nodules. Subsequent thoracic CT demonstrated multiple bilateral pulmonary nodules (Figures 3-4). A CT pulmonary angiography (CTPA), performed following respiratory deterioration, showed nodules with internal cavitation (Figures 5-6). In the setting of *K. pneumoniae* bacteremia and deep genitourinary infection, these findings were considered highly suggestive of SPE. The difference in sensitivity between chest radiography and CT limits conclusions about the exact timing of nodule development.

**Figure 3:**
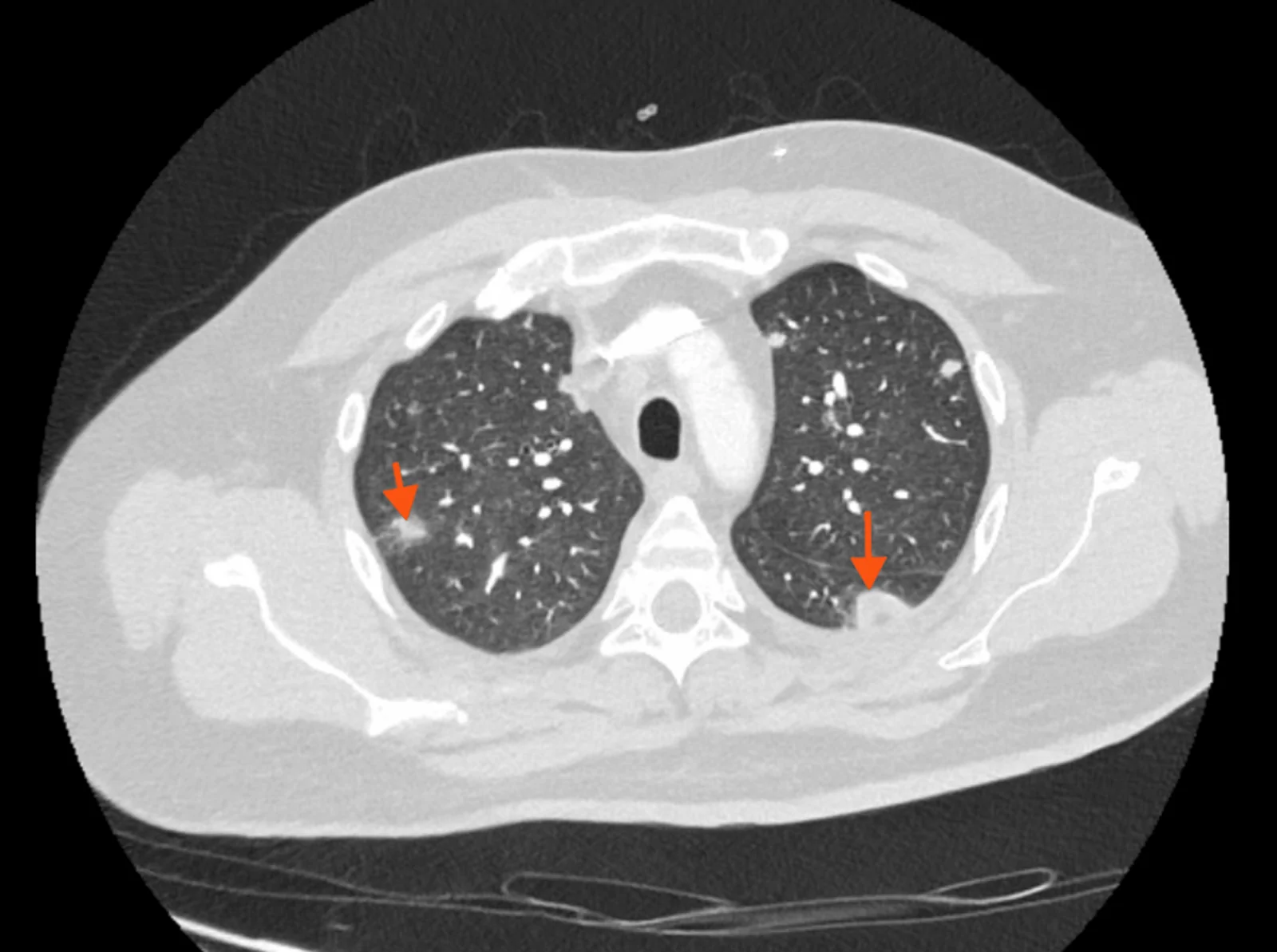
Axial thoracic CT demonstrating bilateral peripheral pulmonary nodules (orange arrows)

**Figure 4:**
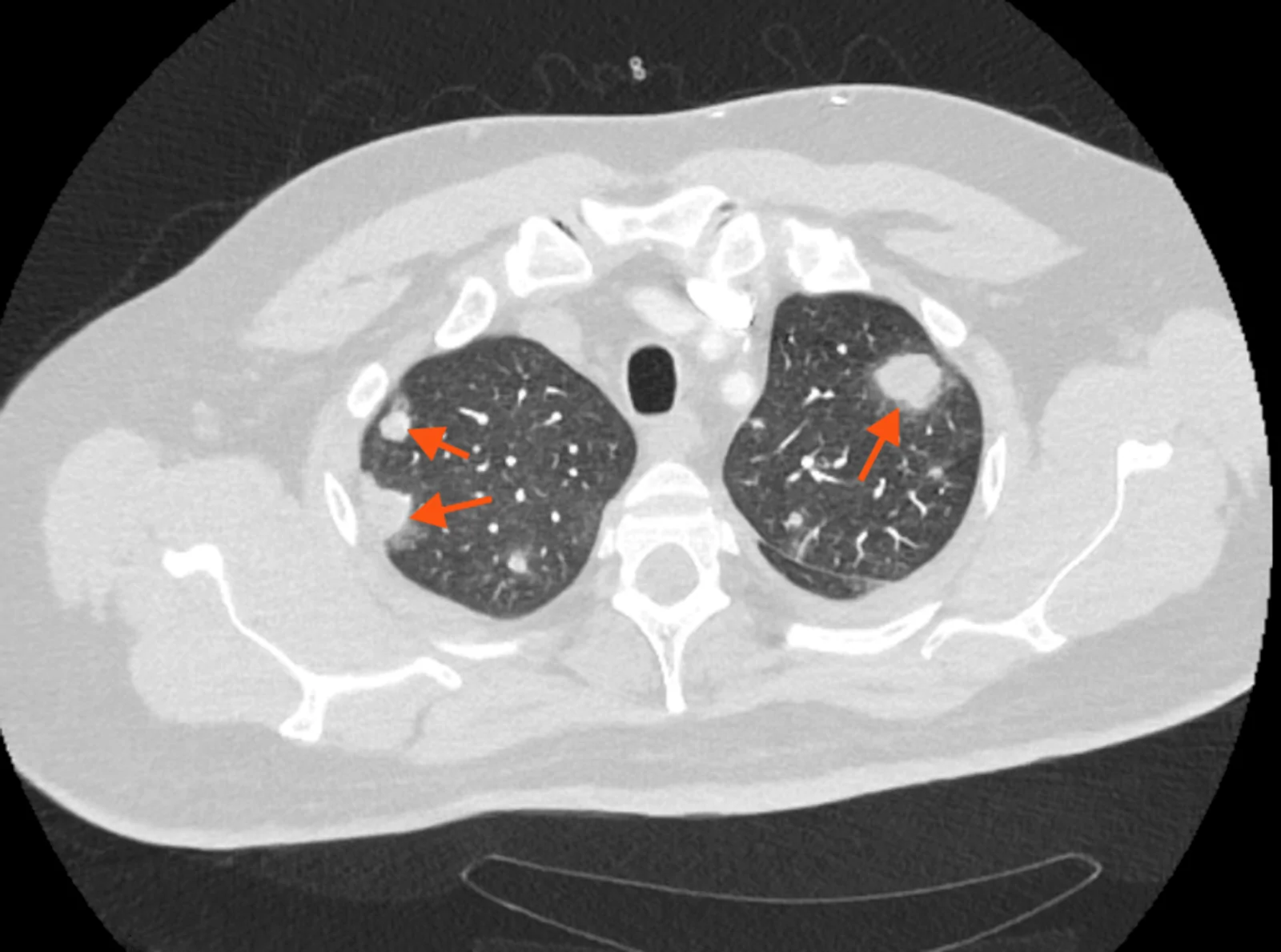
Axial thoracic CT demonstrating additional bilateral pulmonary nodules (orange arrows)

**Figure 5:**
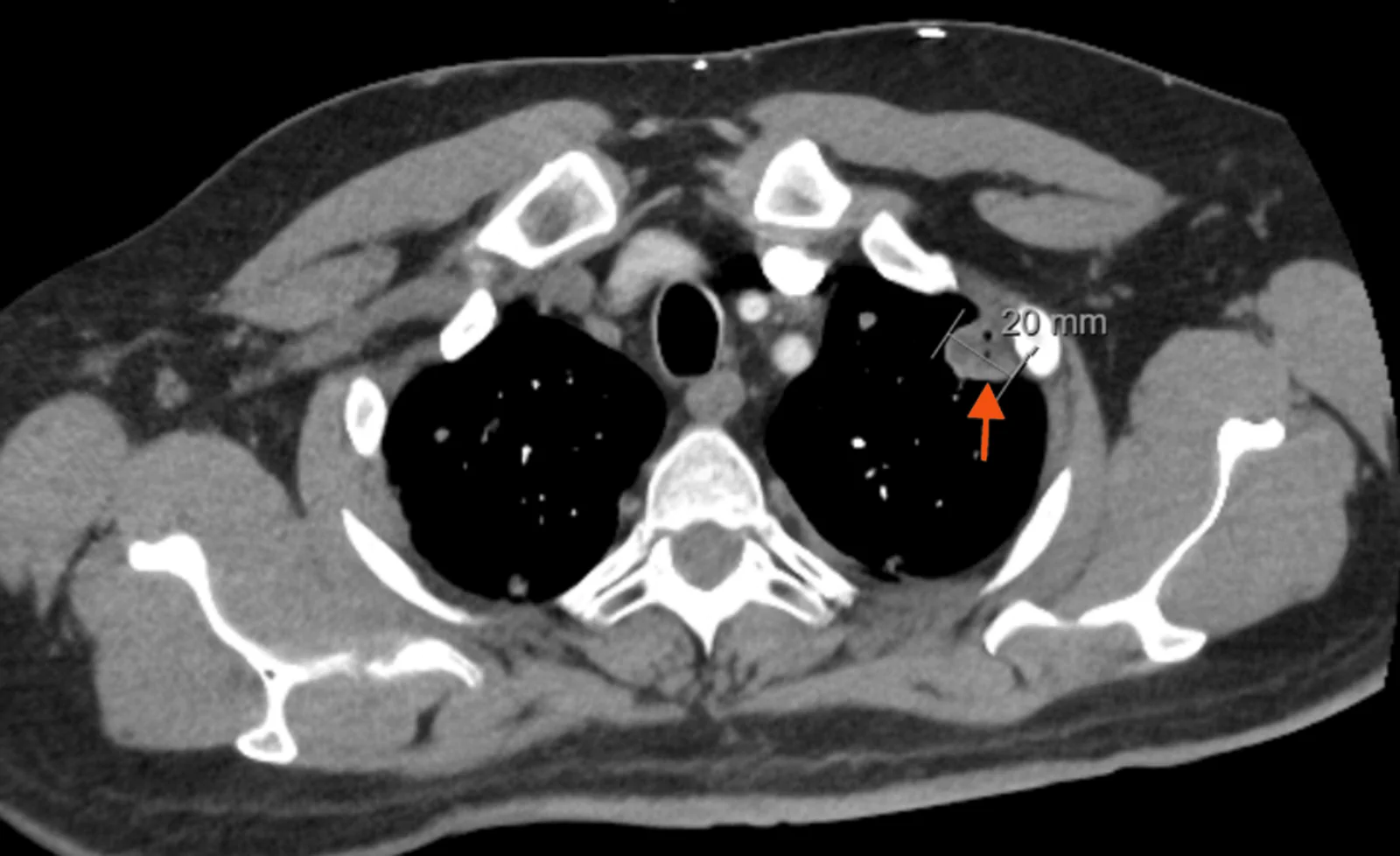
The CTPA showing a pulmonary nodule with internal cavitation (orange arrow), compatible with a septic embolic lesion in the clinical context. CTPA: Computed tomography pulmonary angiography

**Figure 6:**
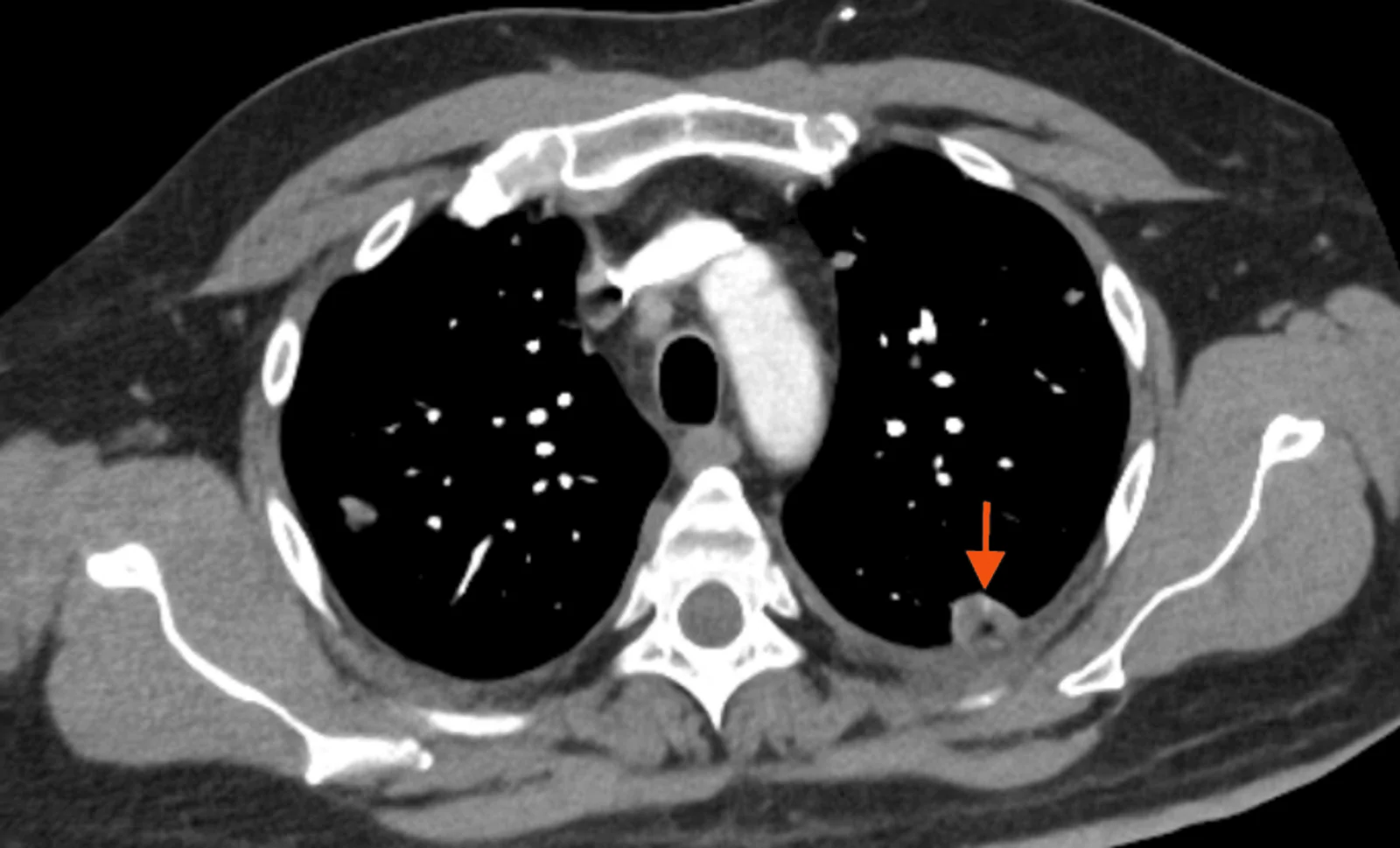
The CTPA showing an additional cavitating pulmonary nodule (orange arrow). CTPA: Computed tomography pulmonary angiography

An echocardiogram was requested but was not completed before transfer to another hospital for drainage of the prostatic abscess. No cardiac murmur was documented on examination. No subsequent echocardiographic result was available in the information reviewed for this report.

The prostatic abscess was drained for source control. The reported treatment course included two weeks of intravenous antibiotic therapy, together with management of hyperglycemia. Following drainage and treatment, fever and dyspnea resolved, and supplemental oxygen was discontinued. The WBC count decreased to 10 × 10⁹/L (reference range: 4-10 × 10⁹/L), and CRP decreased to 70 mg/L (reference range: ≤5 mg/L). Follow-up pelvic MRI demonstrated resolution of the prostatic abscess and significant reduction of prostate size as compared to the previously documented enlarged prostate (Figure 7).

**Figure 7:**
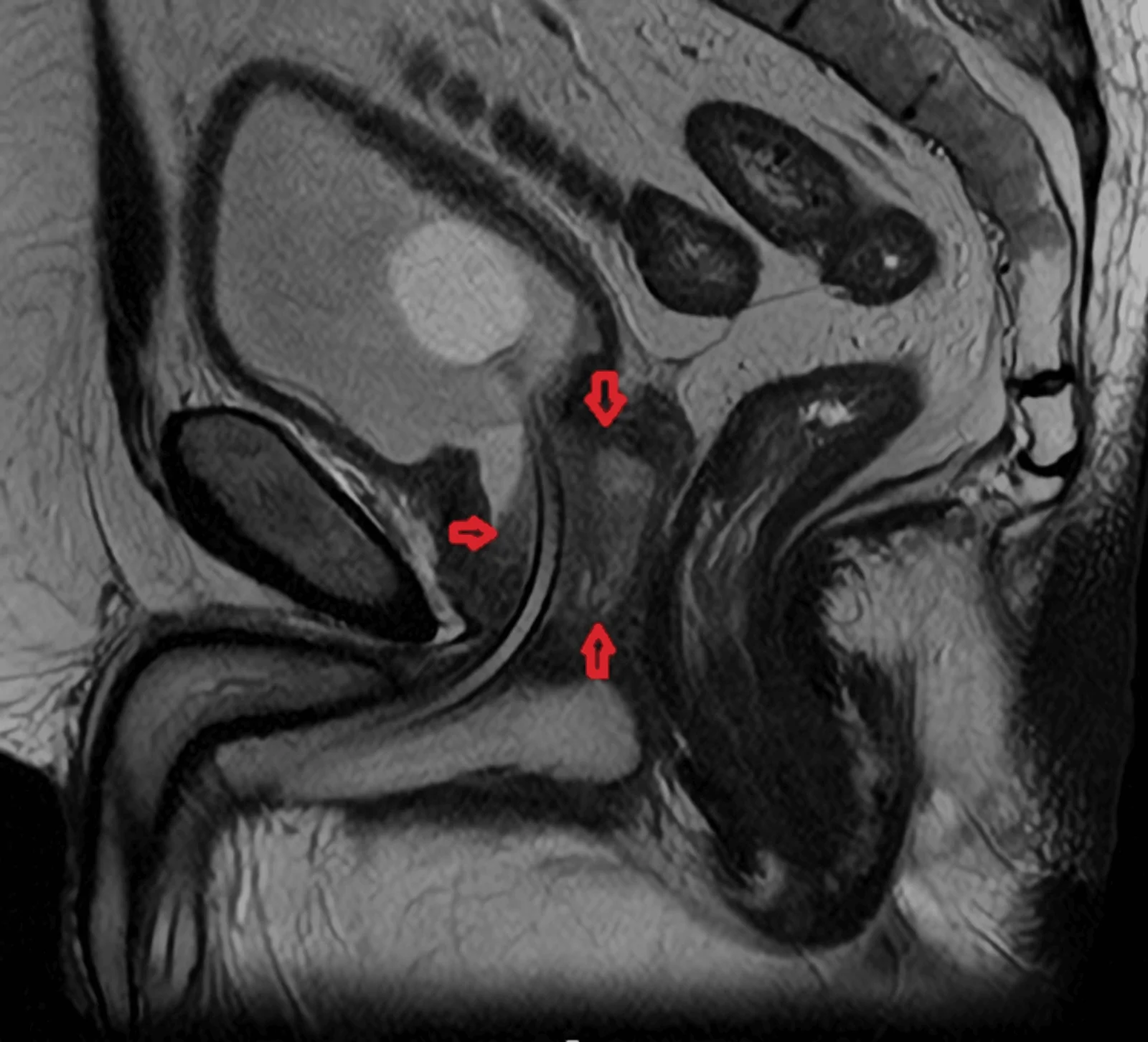
The MRI of the pelvis showing resolution of the prostatic abscess with significant reduction of prostate size (red arrows).

## Discussion

This case illustrates extensive prostatic infection associated with *K. pneumoniae* bacteremia, renal/perirenal infection, and probable septic pulmonary embolic disease in a patient with diabetes. Its educational value lies in the need to investigate a deep infectious focus when systemic illness does not improve with initial antimicrobial therapy and to reassess the extent of infection when respiratory symptoms develop.

Imaging established the presence and extent of the prostatic abscess. A CT identified suspicious prostatic abnormalities, while MRI demonstrated extensive involvement. Published reports describe the role of cross-sectional imaging and drainage in the management of prostatic abscess [2,3,12]. The improvement following drainage and antimicrobial therapy in this patient supports the clinical importance of source control, although the contribution of each treatment cannot be assessed separately.

The pulmonary nodules and cavitation were compatible with SPE in the context of bacteremia and a deep infectious focus. These features are described in both imaging studies and clinical series [6-10,13]. However, they are not specific to SPE. Other considerations include multifocal pulmonary infection, cavitating malignancy, and inflammatory disorders. The acute clinical course and improvement with treatment favored an infectious explanation.

The initial chest radiograph and later CT examinations should not be interpreted as equivalent measures of disease burden. A CT may identify embolic lesions that are not apparent on chest radiography [6,7]. Accordingly, the imaging supports recognition of pulmonary involvement during the admission but does not by itself establish that all nodules formed within three days. Serial imaging using comparable modalities and documented examination dates would be required to substantiate that claim.

Although the genitourinary infection was a plausible source, the prostate cannot be established as the sole origin of the pulmonary lesions. Concurrent pyelonephritis and a perirenal collection complicate source attribution. In addition, echocardiography was not completed before transfer. The absence of an audible murmur does not exclude infective endocarditis; therefore, an endocardial source remained unexcluded. The pulmonary diagnosis is best described as probable SPE rather than microbiologically or histologically confirmed embolic disease.

*Klebsiella pneumoniae* is associated with invasive abscess-forming infections, and a prior report described prostatic abscess complicated by SPE and meningitis [4,5,11]. The clinical pattern alone does not establish a hypervirulent strain. No strain-typing or virulence-testing result was available for this case, and hypervirulence cannot be inferred from the organism identification alone.

A previous case report described *K. pneumoniae* prostatic abscess complicated by septic pulmonary emboli and meningitis [11]. The present case therefore does not represent a unique association between prostatic abscess and septic pulmonary embolic disease. Its clinical relevance lies instead in the extensive intraprostatic involvement, concurrent renal and perirenal infection, development of cavitating pulmonary nodules during persistent systemic infection, and the diagnostic uncertainty regarding the precise source of the pulmonary lesions. These features emphasize the importance of reassessing the extent and source of infection when clinical improvement does not occur with initial antimicrobial therapy.

The available case documentation did not specify the antibiotic agents, doses, or susceptibility results; the drainage technique or drainage culture findings; or whether oral therapy followed the reported intravenous course. The relationship of the two-week intravenous course to the date of drainage was also not established. Repeat blood culture results were unavailable, so persistent bacteremia and the timing of bloodstream clearance could not be confirmed. The HbA1c, exact imaging dates, the interval to follow-up MRI, and follow-up thoracic imaging were not available. These omissions limit assessment of treatment duration, radiological progression, and longer-term pulmonary outcome. Echocardiographic exclusion of an endocardial source was not documented.

## Conclusions

Prostatic abscess should be considered in patients with diabetes and *K. pneumoniae* bacteremia who remain systemically unwell despite appropriate antimicrobial therapy. New respiratory symptoms accompanied by peripheral cavitating pulmonary nodules should prompt consideration of septic pulmonary embolic disease and evaluation for potential infectious sources, including endovascular infection. In this case, drainage and antimicrobial therapy were followed by clinical improvement and MRI-confirmed resolution of the prostatic abscess. The pulmonary findings were suggestive of probable septic pulmonary embolic disease, but the precise source of the embolic lesions could not be established.
